# Host, pathogen and the environment: the case of *Macrobrachium rosenbergii*, *Vibrio parahaemolyticus* and magnesium

**DOI:** 10.1186/s13099-016-0097-1

**Published:** 2016-04-25

**Authors:** Suma Tiruvayipati, Subha Bhassu

**Affiliations:** Department of Genetics and Molecular Biology, Institute of Biological Sciences, Faculty of Science, University of Malaya, 50603 Kuala Lumpur, Malaysia; Centre of Biotechnology for Agriculture (CEBAR), University of Malaya, Kuala Lumpur, Malaysia

**Keywords:** *Macrobrachium rosenbergii*, *Vibrio parahaemolyticus*, Magnesium, GbpA, Chitin

## Abstract

*Macrobrachium rosenbergii* is well-known as the giant freshwater prawn, and is a commercially significant source of seafood. Its production can be affected by various bacterial contaminations. Among which, the genus *Vibrio* shows a higher prevalence in aquatic organisms, especially *M. rosenbergii*, causing food-borne illnesses. *Vibrio parahaemolyticus*, a species of *Vibrio* is reported as the main causative of the early mortality syndrome. *Vibrio parahaemolyticus* infection in *M. rosenbergii* was studied previously in relation to the prawn’s differentially expressed immune genes. In the current review, we will discuss the growth conditions for both *V. parahaemolyticus* and *M. rosenbergii* and highlight the role of magnesium in common, which need to be fully understood. Till date, there has not been much research on this aspect of magnesium. We postulate a model that screens a magnesium-dependent pathway which probably might take effect in connection with *N*-acetylglucosamine binding protein and chitin from *V. parahaemolyticus* and *M. rosenbergii*, respectively. Further studies on magnesium as an environment for *V. parahaemolyticus* and *M. rosenbergii* interaction studies will provide seafood industry with completely new strategies to employ and to avoid seafood related contaminations.

**Electronic supplementary material:**

The online version of this article (doi:10.1186/s13099-016-0097-1) contains supplementary material, which is available to authorized users.

## Background

*Macrobrachium rosenbergii* is a freshwater prawn species of which there is a considerable production range when compared to *Macrobrachium nipponense* (information sourced from http://www.fao.org/fishery/culturedspecies/Macrobrachium_rosenbergii/en). Seafood is affected by several bacteria, and the major factors affecting bacterial survival in sea water are: absence of required nutrients, presence of toxic substances in sea water, presence of bacteriophages, adsorption of bacteria and their sedimentation, the harmful action of the sunlight, utilization of bacteria as food by not only protozoa, but other predators and competitive, antagonistic effects of the microorganism [1].

There are a wide range of bacteria such as *Vibrio cholerae*, *Escherichia coli* 0157:H7, Shigella, *Campylobacter jejuni*, Leptospirosis, Salmonella, *Helicobacter pylori*, Legionella and the *Mycobacterium avium* complex reported from contaminated water (information sourced from http://www.cdc.gov/healthyswimming) [2, 3]. However, mostly *Vibrio* species are pathogenic to marine organisms. Previously, pathogenicity of *Vibrio anguillarum*, *Vibrio anginolyticus*, *Vibrio panaeicida*, *V. vulnificus*, *Vibrio harveyi*, and *Vibrio salmonicida* was observed in the population of fish and other marine organisms such as eel [4, 5]. Those associated with coral reef bleaching were *Vibrio campbellii*, *Vibrio shiloi*, *V. harveyi* and *Vibrio fortis*. These *Vibrios* are a real cause of concern especially in the aquaculture industry [6].

In terms of aquatic food borne diseases, various virulence factors highlight *Vibrio vulnificus*, *Vibrio parahaemolyticus*, and *V. cholerae* considerably important. The factors primarily include the capsular polysaccharide, lipopolysaccharide, cytotoxins and flagellum [7, 8]. While *V. parahaemolyticus* and *V. cholerae* are mostly related to oysters, causing gastroenteritis [9]. *Vibrio vulnificus* was observed to cause primary septicemia not only in marine populations [10], but also in humans. Most cases of infection were reported due to the consumption of seafood [11], especially shellfish [12–22]. *Vibrio vulnificus* was reported to have caused high fatality rates due to its invasiveness associated with soft-tissue infection and severe sepsis [8]. This species was reported in an encapsulated form, which most commonly occurs in clinical isolates rather than environmental isolates [17].

Other species such as *Vibrio fluvialis*, *Vibrio mimicus*, *Vibrio alginolyticus*, *Photobacterium damsel* (*Vibrio damsela*), *Vibrio metschnikovii*, *Vibrio cincimnatiensis*, *Vibrio fuenisii* and *Vibrio hollisae* are also known to be pathogenic [23, 24]. These can cause severe infections to environmental specimens as well as human. *Vibrio parahaemolyticus* in particular was identified as a cause of food-borne illnesses [25], and is associated with the consumption of crab [26]. It was also associated with seafood contamination ranging from crustacean, molluscan shellfish to the giant water prawn. *Vibrio parahaemolyticus* was previously studied of its infection in *M. rosenbergii*, with the latter’s expressed immune genes [27]. Studies even reported *N*-acetylglucosamine binding protein in other species of *Vibrio*. It was shown to have the ability to bind chitinaceous structures such as the outer covering of crustaceans [28–30]. Several studies on GbpA in relation to *Vibrio* show GbpA as an attachment factor to the host chitin (the exoskeleton of crustaceans is called a carapace and consists of chitin) [28, 30, 31]. There are no studies yet on the aspect of GbpA in *V. parahaemolyticus* in particular, and its attachment to chitin of *M. rosenbergii*. The yet unmapped factors of *V. parahaemolyticus* are involved in triggering bacteria to possibly enter the prawns (*M. rosenbergii*) which are our concern in the present review article.

The farming of *M. rosenbergii* in modern times started in the early 1960′s (http://www.fao.org/docrep/005/y4100e/y4100e04.htm#P193_35649). It was during this time, *M. rosenbergii* require brackish water conditions for its survival, though being found as a freshwater prawn [32]. However, *V. parahaemolyticus* was observed in both brackish and fresh water [33]. From the above, the water conditions required by the prawn and bacteria appear quite similar. Hence, the term “conditions for growth” which precisely defines the effect of environmental factors cannot be ruled out in such studies. Therefore, the implication of dealing with host and the pathogen in connection with the environment is conferred by considering *M. rosenbergii,V. parahaemolyticus,* and magnesium. Based on this, a preliminary designed experiment was conducted by us in our lab at University of Malaya and the work is currently under communication as a research article. Our current review hypothesis the possible rhythmic roles that *V. parahaemolyticus* GbpA and *M. rosenbergii* chitin play in the presence of a magnesium environment which could indeed be very useful in not only farming of prawn, but also in future aquaculture research.

### *Macrobrachium rosenbergii* lifecycle

*Macrobrachium rosenbergii* resides in the tropical environments of the freshwater (http://www.fao.org/docrep/005/y4100e/y4100e04.htm#P193_35649), but is influenced by the areas of brackish water. The female prawn bears a gelatinous mass underneath and between the fourth pair of its walking legs. It is here that the male prawn deposits the sperm. After a few hours of mating, eggs are laid and are fertilized by the sperm. “Berried Females” is the terminology used for females carrying the eggs [34]. During the course of embryo development, the eggs remain constantly adhered to the female. It is during this time that the females migrate towards estuaries as the larvae cannot survive in fresh water for more than 2 days. The eggs hatch in brackish water where the salinity ranges from approximately nine parts per thousand (ppt) to 19 ppt [34], and they exist as free-swimming larvae at this stage.

The changes in phase from a larval to a post larval stage is very crucial in a prawn’s life cycle as it grows by the process of moulting (http://www.thefishsite.com/articles/464/moulting-and-behaviour-changes-in-freshwater-prawn/). It undergoes around 11 moults to transform into post larvae. These moults represent a process of metamorphosis. This stage is a critical part of a prawn’s life cycle as the old exoskeleton is replaced by a new soft exoskeleton underneath. It is here that the *M. rosenbergii* absorbs water into the tissue to increase in size (http://www.thefishsite.com/articles/464/moulting-and-behaviour-changes-in-freshwater-prawn/). Hence, the environmental conditions play a significant role in *M. rosenbergii* to enhance its ability to grow into an adult or to alter its chances of survival.

### *Vibrio* genomes and distribution

*Vibrios* are widely distributed in marine environments and are easily adaptable to changes. Hence, these bacteria are considered significant for elucidating correlation between genome evolution and adaptation [35]. 16S rRNA sequence is the basis on which the *Vibrio* species are largely classified within the Vibrionaceae family. To establish the DNA patterns of epidemiological interest, which are associated with the pathogenicity of the strain and to record correlation of diseases among bacteria with specific strains, serotyping was identified as one of the useful markers [36]. Further, the distribution and emergence of pathogenic bacterial strains, the prediction of events [37, 38] through construction of models, and the identification of evolutionary relationships were also done by multi-locus sequence typing/analysis, serogroup association and comparative genomics [39]. For example, with the potential pathogenicity of *V. cholerae, V. parahaemolyticus*, and the association of their serogroups, the specificity of the serogroups was correlated [36, 40, 41]. Studies on comparative genomics of *Vibrio* dealt with the phylogeny of 86 species of *Vibrio* and nine house-keeping genes primarily targeting biodiversity and genome evolution [42]. However, comparative genomic analysis among both the pandemic and non-pandemic Vibrios distributed worldwide has to glean into the bacterial adaptation, evolution as well as antibiotic resistance. Such studies have dealt with the role of integrons in *Vibrio* species for which genes comprise of approximately 1–3 % of the genome [43], genome plasticity shaped by HGT and comparative analysis of pandemic and non-pandemic species [44, 45]. Considering the above studies, the distribution of *Vibrio* in different environmental conditions could be a significant factor responsible for its evolution, resistance, virulence and adaptation.

## Growth conditions of the host and pathogen

### *Vibrio parahaemolyticus* growth conditions

*Vibrio parahaemolyticus* causes wound and nosocomial infections, abdominal pain, diarrhoea, nausea, vomiting and gastroenteritis [26, 46–48].

#### Temperature and growth

*Vibrio parahaemolyticus* is a Gram-negative bacterium which is curved and rod-shaped. It is a non-spore forming bacterium whose high motility is due to a polar flagellum. By a mechanism called swarming, these bacteria migrate across semi-solid surfaces [49] with the help of their lateral flagella. Throughout the world, inshore marine waters are the primary area where the distribution of *V. parahaemolyticus* is in abundance. It is mostly an inhabitant of estuarine marine water. The effect of seasons on *V. parahaemolyticus* has reported that *V. parahaemolyticus* in a small number was isolated from among sediment samples of marine water, but was not detected during the period of winter (i.e., November–March) in the Chesapeake Bay seawater [50]. *Vibrio parahaemolyticus* is proposed to multiply when there is an increase in temperature i.e., by re-introduction of the microorganism into the sea water or by living in the marine sediments throughout the winter [51].

The temperature ranging from 35 to 39 °C [52] are the optimal conditions for the growth of *V. parahaemolyticus*. Though the doubling time of *V. parahaemolyticus* is as little as 5 min [53], under optimal conditions this organism has a generation time of less than 20 min. Hence, *V. parahaemolyticus* is most prevalently observed in a suitable environment in the course of the warm season. In peaking summer, it causes food borne outbreaks as it exhibits mesophilism [54, 55]. Though the count of *V. parahaemolyticus* in seafood which is freshly harvested are rather lower than the dose of infection predicted [56], the rapid multiplying ability of this bacterium at suitable temperatures shows its presence in food, is enough to cause a disease.

#### Salinity

*Vibrio parahaemolyticus* has an important need for its multiplication and living conditions, which is salinity. *V. parahaemolyticus* encounters salinity concentrations in the marine environment typically ranging between 0.8 and 3 % [57]. With optimal levels ranging between 1 and 3 %, *V. parahaemolyticus* can thrive very well in different concentrations of sodium chloride, i.e., between 0.5 and 10 % based on laboratory studies [58].

#### Metals

Apart from salinity, the capacity of the organism to utilize, tolerate and thrive in marine conditions is affected by several different concentrations of metal ions present. *V. parahaemolyticus* isolates are found to survive in 300 mM magnesium (approximately 73,941 ppm), a condition which is considered as toxic to various other microorganisms. This is an example from severely polluted coastal waters in some parts of India [59]. *Vibrio parahaemolyticus* survival rates under several conditions can be improved by the increase in its ability to utilize magnesium. A 5.5 kb plasmid in the bacterium is said to carry genes responsible for bacterial resistance to increased magnesium concentrations [59]. Injured or thermally treated *V. parahaemolyticus* cells show increased uptake of magnesium, which indicates a possible higher requirement for magnesium not only for the stability and repair [60] of its ribosomes, but also its cell membrane.

*Vibrio parahaemolyticus* capability to survive magnesium or any metal ion at high concentrations out-competes other microorganisms of seawater for its own survival and growth in the presence of these ions.

### *Macrobrachium rosenbergii* growth conditions

The optimal range for prawn larvae to survive is 28–31 °C. It was observed that a salinity of <10 % ppt would be ideal for hatcheries for freshwater prawn [32]. Though calcium shows an important role in the formation of the exoskeleton (http://www.thefishsite.com/articles/464/moulting-and-behaviour-changes-in-freshwater-prawn/), it is the conditions which are favourable for the “survival” of larvae which stands of primary importance. There were reports which described magnesium as an important component in the environment for prawn survival. One such previous literature explains the requirement of the magnesium in juvenile prawns [61]. A recent article [62] describes the effects of salinity with the use of artificial sea water. Here, it clearly explains the role of magnesium in the survival rates of post larvae. Taking an example of the effect of an acidic environment in the presence of aluminium, an increase in the magnesium ion (Mg^++^) was observed showing its importance in the survival stages of the post larvae [63]. The composition of water which are good for prawn hatcheries are said to be 10–27 parts per million (ppm) magnesium in freshwater, 1250–1345 ppm magnesium in seawater and 460–540 ppm magnesium in brackish water [32].

These features and conditions show how important is the magnesium ion for the survival of larvae which undergo a very critical “moulting stage” before reaching the post-larval stage.

### *N*-acetylglucosamine-binding protein, chitin and *Vibrio parahaemolyticus*

**Fig. 1:**
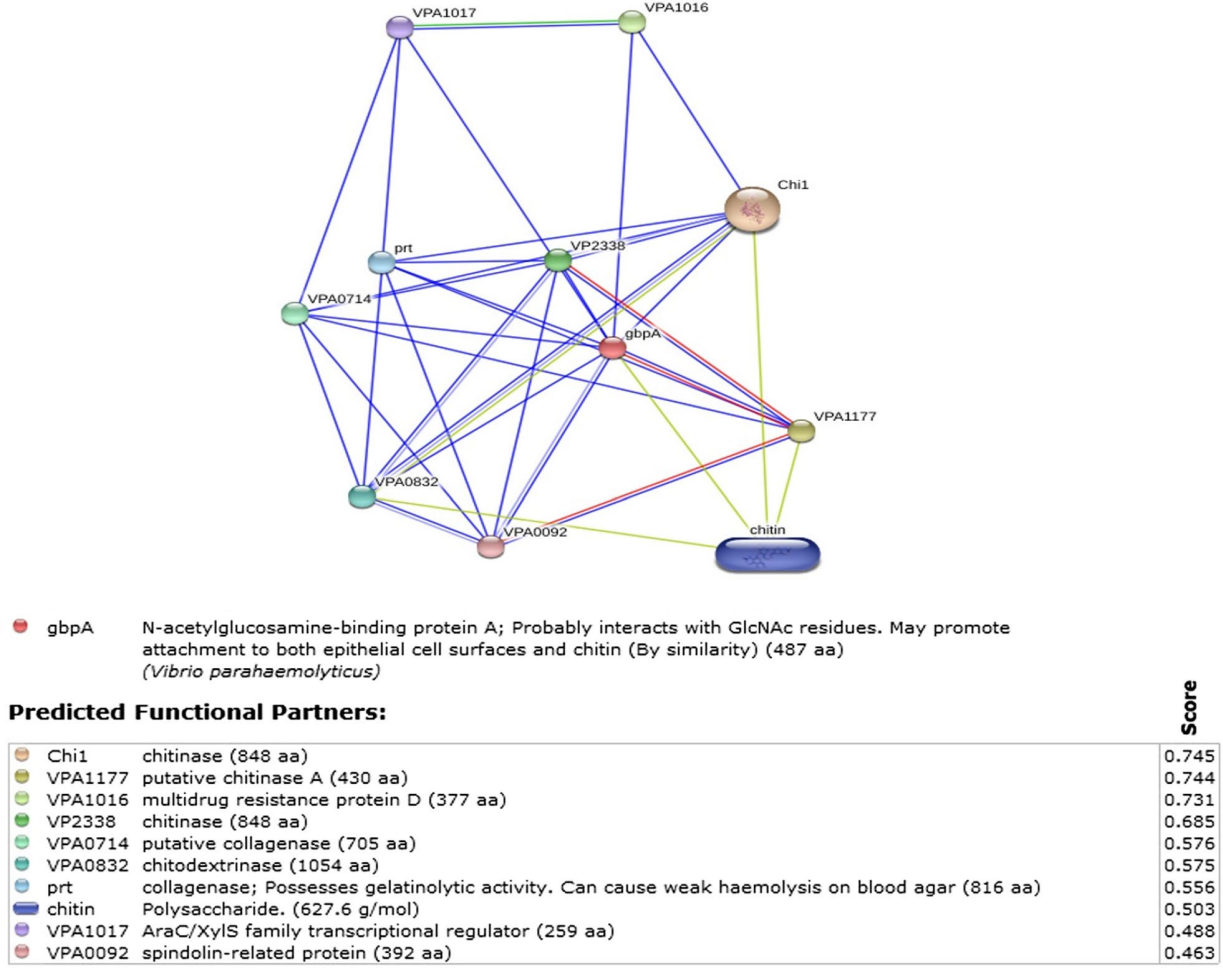
*Vibrio parahaemolyticus* gbpA protein (GbpA) interactions with chemicals on the STITCH 3 database. The predicted functional partners on the STITCH 3 database are most importantly chitinase, collagenase, multidrug resistance protein D and chitinodextrinase. Chitin is also observed as one of the predicted functional partner to GbpA, which supports GbpA’s possible interactions with chitin

*N*-acetylglucosamine-binding protein was reported in *Vibrio cholerae* [30, 31] with its property to bind to epithelial cell surfaces and chitin in the host’s exoskeleton. The probable interactions of the *V. parahaemolyticus* GbpA (Additional file 1) was estimated from STITCH 3 [64] interaction database as shown in Fig. 1. Figure 1 even shows the protein-chemical interactions of GbpA with chitin. The role of prawn chitin was previously studied with the ecology of toxigenic *V. cholerae* and cholera transmission [29, 65–70]. In few studies it was even observed that *V. parahaemolyticus* gets absorbed onto chitin particles and was dependent on several factors such the ions and the pH of seawater [71]. Whereas, this was not observed in other bacteria such as *E. coli* or *Pseudomonas flourescens* [71]. This shows how significant environment could be for bacteria to attach to the chitin of prawn, i.e., in the present scenario *V. parahaemolyticus* to the carapace of *M. rosenbergii*. The effect of GbpA attachment to chitin could be of potential hypothetical interest as previous studies showed that a type IV pili of *V. parahaemolyticus* mediates the attachment to chitin [72]. An increase in the bacterial count in the presence of both chitin flakes and phosphate-buffer saline [73], but not in the presence of *N*-acetylglucosamine, starch and casein could probably support the link between the host and pathogen. This is explained with GbpA in relation to chitin in the presence of environmental magnesium further in the review. Bacteria such as *V. fluvialis*, *V. parahaemolyticus*, *V. alginolyticus*, *V. mimicus*, *Listonella anguillarum* and *Aeromonas hydrophila* were found to be capable of utilizing chitin as a sole source of nutrient in river as well as marine waters [74]. This study shows, there could be probable interactions between GbpA and chitin of the host and pathogen. All these above mentioned factors could support the importance of GbpA and chitin as biomolecular counterparts from the bacteria and prawn, respectively.

### *Macrobrachium rosenbergii* and *V. parahaemolyticus* appear to share a common magnesium environment

*Vibrioparahaemolyticus* has several virulence factors with which it can survive aquatic organisms, especially the giant fresh water prawn, *M. rosenbergii* [75].

The growth conditions of *M. rosenbergii* in the environment can be studied in depth to understand the adaptation correlation of *V. parahaemolyticus* to *M. rosenbergii*. Studies show that *M. rosenbergii* survival in different media compositions was observed with variations in NaCl, KCl and MgCl_2_ + MgSO_4_ [54].

The fertilization envelope of shrimp eggs was observed to thin, when there is a depletion in calcium and magnesium [76]. Embryos in their early stages were shown to require optimal levels of medium including MgCl_2_ + MgSO_4_ for their proper development [77]. The role of magnesium ion in the normal hatching rate or the newly hatched larvae was not shown to be significant [77], but its importance in prawn survival was observed [62].

There are various resistance factors which *V. parahaemolyticus* carry such as: cobalt, zinc, cadmium, and chromium resistance genes [78]. This can also explain its possible survival rate with *M. rosenbergii*, which could have been exposed to toxic substances during its life cycle [79, 80]. During the course of evolution, the bacteria must have acquired these resistance genes on prolonged exposure while surviving together with the host, which is *M. rosenbergii*. The most interesting factor is the tolerance of *V. parahaemolyticus* unlike other bacteria to higher concentrations of magnesium, and its growth under iron-limiting conditions which appears directly proportional to conditions of the prawn larvae survival as mentioned earlier. Various studies on the importance of magnesium in *Vibrio* species support its significance as an environment, which was observed in one scenario where magnesium sulfate could regulate luminescence in *Vibrio fischeri* [81], while in the other, magnesium had a very high impact in promoting flagellation in *Vibrio* [82]. Previously, research was done to check the effect of magnesium ion in protein secretion by magnesium-resistant bacterial strains [59] which indeed shows that magnesium cannot be ruled out in studies on *Vibrio*. Studies even highlighted that the growth of *V. parahaemolyticus* under iron limiting conditions was when the bacteria survived high concentrations of magnesium [83].

**Fig. 2:**
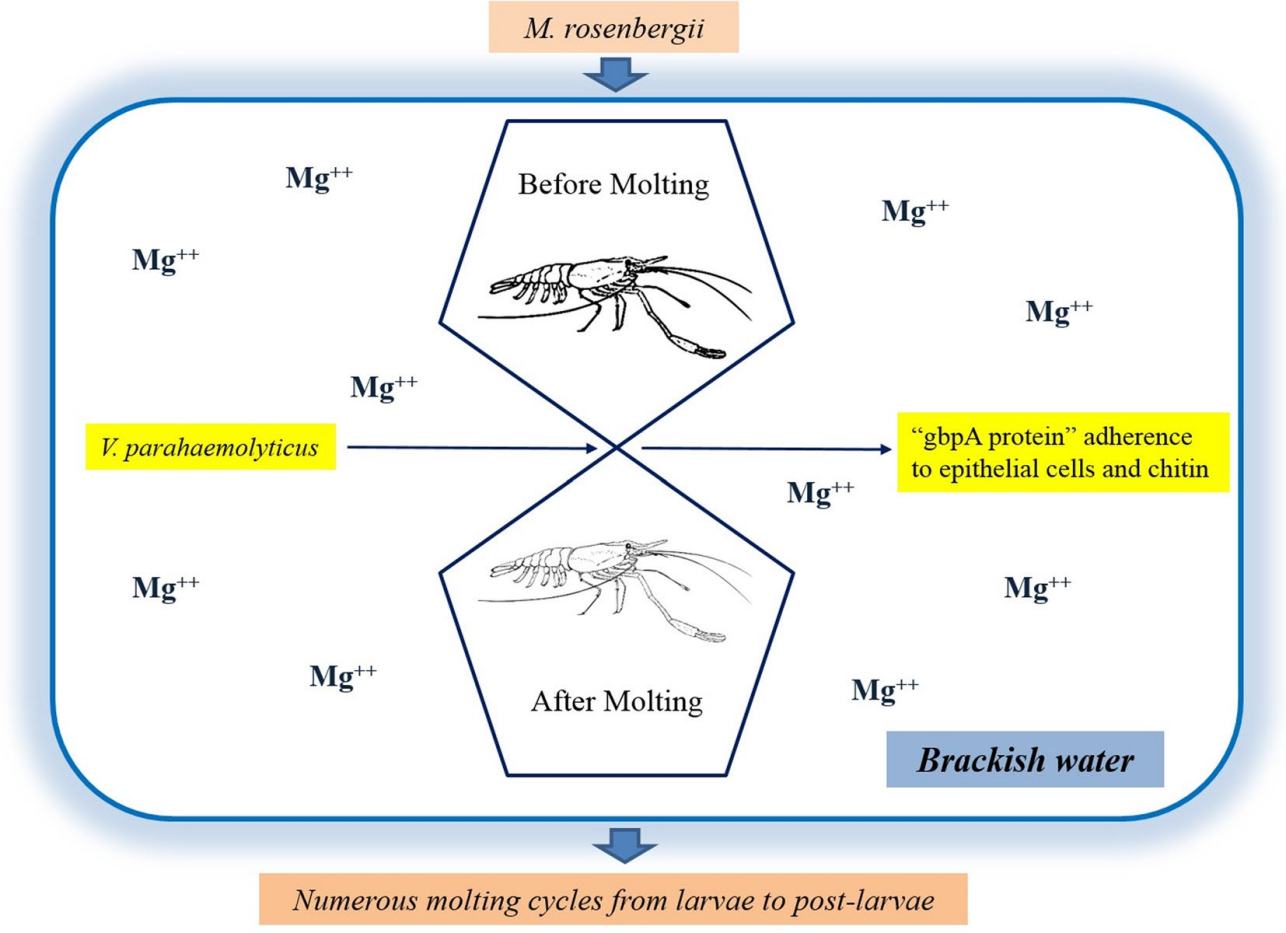
Hypothetical schematic representation of the probable host–pathogen–environment relationship. *Macrobrachium rosenbergii* undergoes a number of moulting stages through the larvae to post-larvae stage. It is during the intervening phase of moulting that the prawn attains a thin exoskeleton. The presence of magnesium in the environment at this phase might influence favourable conditions for *V. parahaemolyticus* GbpA protein (GbpA) to bind/interact with the epithelial cells, especially the sensitive chitinaceous surface of *M. rosenbergii*

Figure 2 is a hypothetical schematic representation which shows magnesium ion as an important link between *V. parahaemolyticus* and *M. rosenbergii*. During the moulting stage of prawn, the prawn often loses a thick moult to regain a transparent exoskeleton (http://www.thefishsite.com/articles/464/moulting-and-behaviour-changes-in-freshwater-prawn/). The figure shows the relation of *V. parahaemolyticus* with the prawn following exuviation in the presence of magnesium. This is conveyed by keeping the magnesium environment constant, i.e., with its levels common to both prawn and bacteria. When a prawn undergoes exuviation, the GbpA of bacteria might probably have greater chances of binding strongly to the sensitive exoskeleton of the prawn. This when compared to the prawn before moulting, its thick exoskeleton might affect the attachment of GbpA to chitin. Here, the binding capacity of GbpA needs to be higher due to a strong layer of chitin containing exoskeleton. This will require further studies to understand the importance of the presence of magnesium to both the host and pathogen.

## Conclusion

With regard to food-borne illnesses, *V. parahaemolyticus* contributes significantly to morbidity worldwide [54].

Apart from controlling the severity of bacterial vigour caused by *V. parahaemolyticus*, strategies to control disease spreading through seafood consumption caused by bacteria adapting to aquatic environments are indeed required and needs more attention. This is because, most human populations worldwide are relying on seafood consumption on a daily basis. There are many aquatic organisms which need to be considered for the control of bacterial infections from spreading. The basis of selecting *V. parahaemolyticus* and *M. rosenbergii* in the current review is because of the widely spreading early mortality syndrome (EMS), which is capable of producing a toxin similar to the cholera which can cause life-threatening diarrhoea [84–86].

We think that the utilization of magnesium ion to check any possible interactions between GbpA and carapace (chitin) of the bacteria and prawn, respectively could probably assist us to understand the significance of a magnesium environment. In the present context, as *V. parahaemolyticus* is dealt in relation with *M. rosenbergii*, a giant freshwater prawn of commercial importance, further research based on the aspect of magnesium ion usage by both the prokaryotic or eukaryotic counterparts could help us understand the contamination strategies better. One such strategy could be tweaking the magnesium levels in order to avoid bacteria from entering aquatic organisms. Our review provides the understanding that maintaining magnesium could be important in order to avoid bacteria from multiplying rapidly to infectious levels. Hence, this could help minimize the risk of contamination in the aquaculture systems which might help control food-borne diseases in the long run.

## Additional file

10.1186/s13099-016-0097-1 Fasta sequence of the *Vibrio parahaemolyticus* GbpA protein.

## Abbreviations

CTXcP: Cholera toxin cP
GbpA: *N*-acetylglucosamine binding protein
