# Supplementary material for: Host, pathogen and the environment: the case of Macrobrachium rosenbergii, Vibrio parahaemolyticus and magnesium
Source: Gut Pathog. 2016 Apr 25;8:15. doi: 10.1186/s13099-016-0097-1 (PMC4843205; doi:10.1186/s13099-016-0097-1)
Supplement: Supplementary file 1 — 10.1186/s13099-016-0097-1 Fasta sequence of the Vibrio parahaemolyticus GbpA protein. [file 13099_2016_97_MOESM1_ESM.doc]

**Additional File 1**

**Fasta sequence of the *Vibrio parahaemolyticus* GbpA protein – The protein sequence was taken from the strain *Vibrio parahaemolyticus* PCV08-7 (**[**http://www.ncbi.nlm.nih.gov/nuccore/AOCL00000000**](http://www.ncbi.nlm.nih.gov/nuccore/AOCL00000000)**)**

**>GbpA**

METKSFPNKSLVALAIASMETSSGVLAHGYVSESNDGVAASRAALCKYPTSDTNERNTNCGAIQYEPQSVEGPDGFPETGPRDGKIASAETARAAALDEQTADRWVKRPIKSGTQTFEWTFTANHVTRDWKYYITKPNWNPNASLSRDSFALNPFCVVDGNMETVQPPKQMETSHQCNVPEREGYHVILAVWDVGDTAASFYNVIDVKFDGDDPVIPEWTQGGQIIPTMETNLKVGDSVYTRVFDQSGENVAYRTELAISNDALTQAKNWSYALASKVNQEQTKLQAGQYSEDKFTPVYGTNPIYLQSNSGLERVEIGYNIETPVPEYSLTVDGLASEYIIATEPTSLDLTLTAEGDLTAELTVYNHHREPLASWTGSIQDGASEQVELTLSKSEPGHHMETLVTRIKDTDGNLVDQQTLDFHLKSEEVTPPPSGEYDFVFPEGLSSYTAGTKVLASDGAIYQCKPFPYSGYCVQWSESATQFEPATGSHWEMETAWDKLN
